# Spatial and temporal determinants of particulate matter peak exposures during pregnancy and early postpartum

**DOI:** 10.1016/j.envadv.2024.100557

**Published:** 2024-06-14

**Authors:** Yisi Liu, Li Yi, Yan Xu, Jane Cabison, Sandrah P. Eckel, Tyler B. Mason, Daniel Chu, Nathana Lurvey, Deborah Lerner, Jill Johnston, Theresa M. Bastain, Shohreh F. Farzan, Carrie V. Breton, Genevieve F. Dunton, Rima Habre

**Affiliations:** a Department of Population and Public Health Sciences, Keck School of Medicine, University of Southern California, Los Angeles, CA, USA; b Department of Epidemiology and Environmental Health, College of Public Health, University of Kentucky, Lexington, KY, USA; c Department of Population Medicine, Harvard Medical School, Boston, MA, USA; d Eisner Health, Los Angeles, CA, USA; e Spatial Sciences Institute, University of Southern California, Los Angeles, CA, USA

**Keywords:** Personal exposure, Fine particulate matter, Primary combustion, Peak exposures, Microenvironment, Time-activity

## Abstract

**Background:**

Fine particulate matter (PM_2.5_) exposure is an important environmental risk for maternal and children’s health, with peak exposures especially those derived from primary combustion hypothesized to pose greater risk. Identifying PM_2.5_ peaks and their contributions to personal exposure remains challenging. This study measured personal PM_2.5_ exposure, characterized primary combustion peaks, and investigated their determinants during and after pregnancy and among Hispanic women in Los Angeles, CA.

**Methods:**

Continuous personal PM_2.5_ exposure, Global Positioning System geolocation, and ecological momentary assessment surveys were collected from 63 women for 4 consecutive days in their 1^st^ trimester, 3rd trimester and 4–6 months postpartum. Based on the shape of PM_2.5_ time-series, primary combustion peaks were identified, characterized (number, duration, area under the curve [AUC]), and linked with locations they occurred in. Zero-inflated generalized mixed-effect models were used to examine the spatial and temporal determinants of PM_2.5_ peak exposures.

**Results:**

A total of 490 PM_2.5_ peaks were identified from 618 person-days of monitoring. Spending an additional minute at parks and open spaces was related to smaller (AUC decreased 3.1 %, 95 % CI: 1.5 %–4.6 %) and shorter (duration decreased 1.7 %, 0.5 %–2.9 %) PM_2.5_ peak exposure. An additional minute in vehicular trips also related to smaller and shorter peak exposure (AUC and duration decreased 2.5 %, 1.2 %–3.7 % and 1.8 %, 1.0 %–2.6 %, respectively). However, an additional minute at industrial locations was associated with greater number (3.6 %, 2.0 %–5.2 %), AUC (1.6 %, 0.1 %–3.2 %) and duration (1.0 %, 0.0 %–2.1 %) of personal PM_2.5_ peak exposure.

**Conclusions:**

This study demonstrates the potential to statistically identify exposure to primary combustion PM_2.5_ peaks and understand their determinants from personal monitoring data. Results suggest that visits to parks and open spaces may minimize PM_2.5_ peak exposures, while visiting industrial locations may increase them in and around pregnancy.

## 1. Introduction

Pregnancy is a critical exposure window to air pollution for both pregnant women and developing babies. An increasing number of studies have revealed adverse health impacts of prenatal and early postpartum air pollution exposure on mothers, fetuses, and infants. For example, exposure to particulate matter less than 2.5 μm in aerodynamic diameter (PM_2.5_) has been associated with gestational diabetes (Cheng et al., 2022), elevated blood pressure (Sun et al., 2020) and postpartum depressive symptoms (Niedzwiecki et al., 2020, Sheffield et al., 2018) in pregnant women. PM_2.5_ has been identified as an important environmental risk factor for adverse birth outcomes (*e.g*., low birth weight, premature birth) (Li et al., 2017, Kloog et al., 2012) and subsequent long-term health in children (*e.g*., asthma, obesity, high blood pressure) (Leon Hsu et al., 2015; Rosa et al., 2020; Rosofsky et al., 2020; Sun et al., 2021).

Most previous prenatal air pollution exposure and health studies, especially large population studies, assessed outdoor air pollution exposure in residential neighborhoods as a proxy of personal exposure to outdoor-origin air pollution. Since people are mobile in time and space, such home address-based stationary exposure assessment could introduce exposure measurement error that may bias and attenuate statistical confidence in health effect estimates (Setton et al., 2011, Evangelopoulos et al., 2020). With the rapidly developed sensing technology, continuous personal air pollution exposure can be measured using wearable sensors or devices that are placed directly on study participants (typically close to the breathing zone) as they go about their usual activities. Such measures of personal exposure with more granularity in time and space can be paired with Global Position System (GPS) to derive context and activities in space and time (Yi et al., 2022) in relation to health measures. These advances allow for identifying timing and sequences of exposures, deriving signatures of specific sources, and exploring alternative exposure metrics (*e.g*., peak exposure or variations instead of the average level of exposure that is mostly used in epidemiological studies (Rabinovitch et al., 2016), which can be more useful to understanding the acute health effects of environmental exposures and to inform potential intervention strategies.

Another advantage of personal air pollution monitoring is the ability to measure the total contribution of all sources impacting what an individual is breathing, including sources that originated indoors (*e.g*., cooking, burning candles), outdoors (*e.g*., infiltration of outdoor pollution indoors), in-transit (*e.g*., on-road traffic emissions), and while conducting a specific activity or occupation. Understanding health effects of outdoor-origin PM_2.5_ (the fraction that is regulated), as most epidemiological studies focused on, is of critical importance to support air quality standard and policy. However, the indoor environment and personal activities contribute significantly to total personal exposure and health, and evidence for more health protective standard or guidelines are warranted (Habre et al., 2022; O’sharkey et al., 2022; Habre et al., 2014; Wheeler et al., 2011; Van Ryswyk et al., 2014).

Primary combustion is a major source of personal PM_2.5_ exposure, including tobacco smoking, cooking, and fossil-fuel combustion (*e.g*., fireplace, diesel exhaust), (Lai et al., 2019; Shang et al., 2019; Chang et al., 2003; Zamora et al., 2018) that is hypothesized to be more toxic compared to the average PM_2.5_ mixture. For example, studies found significant associations of in-utero exposure to PM_2.5_ from traffic and fuel/oil combustion with premature birth (Ottone et al., 2020), gestational diabetes mellitus, and impaired glucose tolerance (Mai et al., 2022). Furthermore, tobacco smoke and second-hand smoke exposure during pregnancy are significantly associated with low birth weight, reduced lung function and fetal mortality (Best et al., 2009). Primary combustion also contributes significantly to periods of time with extremely elevated PM_2.5_ concentrations. While the word “peak” is sometimes used to refer to elevated or maximum PM_2.5_ mass concentrations (regardless of type or source), in this analysis and aerosol science context, it is used to refer to the characteristic peak shape that primary combustion emissions typically exhibit starting with a sharp rise and a gradual decay (Acevedo-Bolton et al., 2011, Ott et al., 2021). Understanding source contributions and health effect of peak exposures from primary combustion, which have been difficult to pinpoint but can be more of a concern for health, is important to inform health-relevant recommendations, especially during pregnancy and early postpartum period to improve maternal and children’s health.

To address the above gaps, this study leverages continuous personal PM_2.5_ monitoring, global positioning systems (GPS) technology, and ecological momentary assessment (EMA, a tool that repeatedly samples participants’ current behaviors and experiences in real time, in their natural environments) to (1) describe total personal PM_2.5_ exposure for Hispanic women during their early pregnancy, late pregnancy and early postpartum, (2) apply and customize a previously developed statistical method to detect and describe PM_2.5_ peak exposure from primary combustion sources, and (3) understand how time-activity and mobility patterns derived from GPS in earlier work impact PM_2.5_ primary combustion peak exposure among Hispanic women during pregnancy and early postpartum.

## 2. Methods

### 2.1. Study design

This analysis is based on the Real-Time and Personal Sampling sub-study (O’Connor et al., 2019) nested within the Maternal and Developmental Risks from Environmental and Social Stressors (MADRES) prospective cohort (Bastain et al., 2021). A total of 65 low-income, Hispanic women in urban Los Angeles drawn from the larger MADRES cohort were recruited into this intensive longitudinal, observational panel study during their 1^st^ and 3^rd^ trimester and at 4–6 months post-partum (*i.e*., a total of three study waves) from 2016 to 2018. The inclusion criteria for this panel study were: (a) < 20 weeks since the date of mother’s last menstrual period at the time of enrollment, (b) 18 years of age or older; (c) singleton pregnancy and (d) Hispanic/Latina ethnicity. Women with current incarceration, HIV positive status, or having physical, mental, or cognitive disabilities were excluded from the study.

During each of the study waves, participants completed four consecutive days (two weekdays and two weekend days) of data collection using innovative personal, continuous data capture strategies (*e.g*., personal PM_2.5_ exposure monitoring, geolocation monitoring from GPS, and EMA). The personal monitoring started on a Thursday or on a Friday based on the participant’s availability All the devices were programmed to start data collecting at 12:00 AM on the first day and turn off at 11:59 PM on the fourth day. Details of the study design can be found in previous publications (Yi et al., 2022; O’Connor et al., 2019). The USC Institutional Review Board approved all study procedures and participants signed an informed consent before enrolling into the study.

### 2.2. Personal PM2.5 exposure

The MicroPEM v3.2A (RTI International, Research Triangle, NC) monitor was used to measure personal exposure to PM_2.5_. The monitor contained a light-scattering nephelometer to record PM_2.5_ mass concentration with a time interval of 1 min. It also incorporated a Teflon filter to collect integrated PM_2.5_ samples for gravimetric analysis, which could be used to further correct the nephelometer readings for known relative humidity artifacts.

**Fig. 1.**
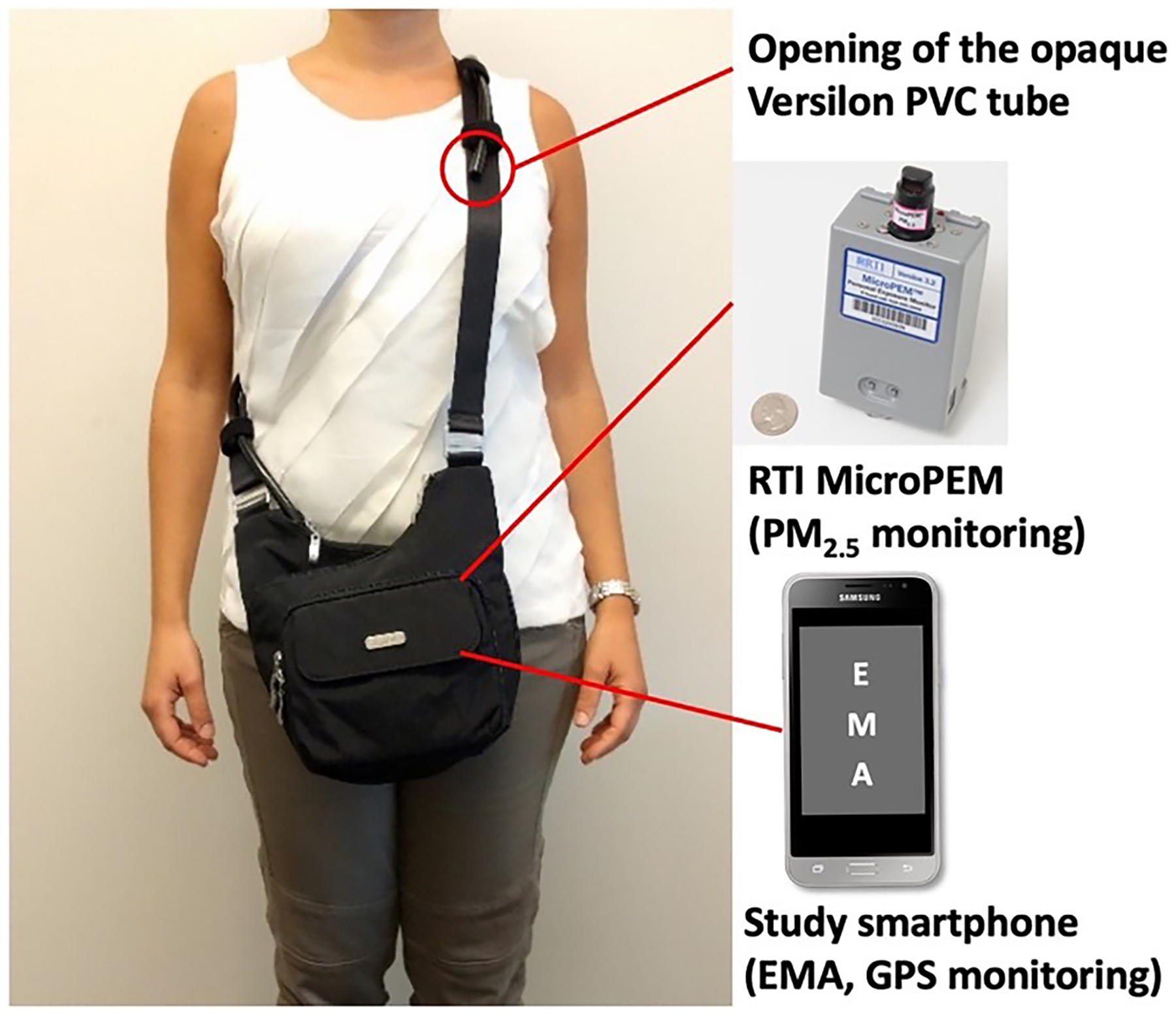
The setup of devices in the MADRES personal monitoring panel study.

During the study, each participant was asked to carry the MicroPEM securely sealed by research staff in a crossbody purse. An opaque Versilon PVC tube was connected to the inlet and fastened along the strap of the purse with its opening beneath the shoulder in the breathing zone (Fig. 1). Participants were asked to wear the purse during their waking hours as much as possible. When the participants expected to stay in one location for an extended period or could not wear the monitor (*e.g*., driving and sleeping), they were instructed to place the purse on a surface close to them but away from humidity (*i.e*., placed in a nearby room when taking showers).

A total of 888,440 min of PM_2.5_ concentration data with 161 filter samples were collected using 6 MicroPEM devices in the study. We applied a mixed effect model to correct the minute-level PM_2.5_ concentrations against the filter samples, where the dependent variable was the gravimetrically weighed, filter-based PM_2.5_ mass concentration and the independent variable was the average of the minute-level PM_2.5_ concentrations during the 4-day sampling period. We included both a random slope and random intercept for each MicroPEM device to account for device-specific differences in response. The correction equation had a R^2^ of 0.952. Finally, all the minute-level PM_2.5_ concentrations were corrected using the equation and used in later analyses.

### 2.3. Personal monitoring compliance

Compliance with wearing the air pollution monitor is an important aspect to help interpret the personal exposure data. Two sources of data were collected to understand and determine participant compliance with personal PM_2.5_ monitoring.

First, a study smartphone with Android operating system was provided to each participant (Fig. 1) and programmed to deliver EMA survey questions up to 5 times per day including questions on compliance with wearing the air monitor (O’Connor et al., 2019). Participants received one EMA survey at a random time during each of the following five time-windows: wake-up – 10 am; 11 am – 1 pm; 2 pm – 4 pm; 5 pm – 7 pm, and 8 pm – bedtime. The normal wake-up and bedtime was collected in a baseline survey for each participant at recruitment. The first survey after waking up asked “Where did you put the air sampling bag when you were sleeping?” with three response options (next to me, same room, and somewhere else). Other day time surveys asked two questions: (1) “Over the past 2 h, how much time did you wear the air sampling bag?” with three response options (all the time, some of the time, and none of the time), and (2) “If you did not wear the air sampling bag sometime over the past 2 h, where did you put it?” with four response options (right next to me, same room but not right next to me, somewhere else, and I wore it all the time).

Second, the MicroPEM device derived continuous wear compliance information based on motion detected by a built-in tri-axis accelerometer. The wear compliance information divided monitor wearing into three categories: wearing the monitor, might not be wearing the monitor, and not wearing the monitor (Rodes et al., 2012).

To combine these two sources of compliance information, we first calculated the median MicroPEM compliance index from minute-level data for each hour. Then, we re-classified wear compliance into three categories by combining microPEM and EMA information as follows: optimal, sub-optimal and worst. Optimal included three conditions: when participants answered that they wore the monitor all the time (EMA), they had the monitor right next to them (EMA), or the MicroPEM showed that they wore the monitor. Sub-optimal included three conditions: when participants answered that they wore the monitor for some of the time in the past two hours, they put the monitor in the same room with them, or the MicroPEM showed that they might be wearing the monitor. The worst compliance category included two conditions: when participants answered that they put the monitor somewhere else or the MicroPEM showed that they did not wear the monitor. Because the participants were guided to put the MicroPEM on a nearby surface or hanging in the same room while sleeping, we used the morning EMA compliance when available for nighttime compliance (from bedtime to wake-up). During the daytime, we used the MicroPEM compliance data when available as it was continuously collected as compared to EMA. When both the EMA and MicroPEM compliance information was not available, the compliance information for that hour was coded as missing.

### 2.4. GPS location and microenvironment characterization

GPS locations were collected to understand participants time-activity patterns and their personal PM_2.5_ exposure in different microenvironments or contexts. To track and record participants’ geolocations, a GPS application (madresGPS app) was developed for this study and was installed along with the EMA application on the study smartphone (Fig. 1) (O’Connor et al., 2019). The madresGPS app recorded geo-locations separately identified by source (GPS and cellular/ Wi-Fi network). Longitude and latitude coordinates and geolocation meta-data were recorded at 10 s epochs and analyzed to detect start and end times of trips and stays as detailed in Yi et al. (2022) Trips were classified as pedestrian walks vs vehicular trips. Stays (including trip origins and destinations) were classified into the following seven contexts: a. home residential location, b. non-home residential location, c. commercial location, d. industrial location, e. facilities, office and education location, f. recreational location, and g. transportation location. Among these, home residential locations were identified as the longest duration of stay during each four-day data collection period and were cross-validated with the self-reported home addresses; non-home residential locations were residential areas other than participants’ home; recreational locations referred to parks and open spaces; and transportation locations included railway and bus stations. Additionally, we classified microenvironments (indoor and outdoor) for each GPS location recorded in the study. Details of the GPS data processing and characterization can be found in a previous publication (Yi et al., 2022).

Since context and microenvironment might be missing when no GPS information was available, further imputation was employed to impute missing data missingness. First, because previous findings from this study showed different time-activity patterns on weekdays and weekends for these participants (Yi et al., 2022), we summarized the most common microenvironment and context (general pattern) during each hour for every person-wave on weekdays and weekend days separately. Then, we used these general patterns to impute any missingness. Hours that still had no data after this step were left as missing in data analyses.

### 2.5. Primary combustion related PM2.5 peaks detection

We analyzed continuous PM_2.5_ mass concentration time-series to identify and derive primary combustion peaks based on their characteristic shape and earlier work by Wallace et al. (2006) This method captured PM_2.5_ peaks with rapid increase at the beginning with gradual decay curves afterwards (at variable rates of decay). Peaks with such characteristic shapes in time-series plot are thought to largely come from primary combustion sources (Acevedo-Bolton et al., 2011; Ott et al., 2021; Wallace et al., 2015; Ott et al., 2014; Cheng et al., 2022). We followed the Wallace et al. algorithm with small modifications that we iteratively refined for our study context to be able to capture peaks we visually identified (tweaks were minor and refined based on visual inspection). First, we subtracted the 5th percentile as the background concentration for each person-wave from the minute-by-minute PM_2.5_ data to minimize the large between-person differences. This approach would guarantee that we did not miss exposure peak for participants with relatively low PM_2.5_ exposure levels. Then, we smoothed the minute-level PM_2.5_ concentration data to 4-minute moving averages to minimize noise in the data which can also be exaggerated under highly variable conditions or in proximity to sources. The running averages were used in all the calculations except for defining the “baseline” (the concentration of PM_2.5_ at the minute when a peak started), which also varied by peak. These three criteria had to be met to define the start (and time of start) of a peak: (1) an increase in the running average by at least 5 mg/m^3^ from 1 min to the next, (2) the observed concentration difference was greater than 8 mg/m^3^, and (3) the ratio between consecutive observations was larger than 0.1. As long as the subsequently measured running averages were at least 7 mg/m^3^ higher than the baseline, the peak continued. The peak ended when (1) its slope changed from negative to positive, (2) the moving average fell below a value 7 mg/m^3^ higher than the baseline, and (3) the moving average was less than 75 % of the baseline value. Aside from determining the start and end of a peak, a peak should also reach a minimum concentration level of 15mg/m^3^ and last more than 5 min. With all the detected peaks, the background concentration (5 % percentile of PM_2.5_ concentrations) for each person-wave was added back to calculate the area under the curve (AUC, corresponding to potential inhaled dose) and the maximum concentrations for each peak.

After identifying all the primary combustion peaks for each person-wave, we merged PM_2.5_ concentrations with identified peaks, peak descriptors (duration, AUC, and maximum concentration), and GPS locations with assigned microenvironment and context at the minute-level.

### 2.6. Statistical analyses

Medians, geometric means (GM), and geometric standard deviations (GSDs) of personal PM_2.5_ exposure were calculated by study wave (1st and 3rd trimesters of pregnancy and 4–6 months postpartum), microenvironment, and context. Number of peaks and their average duration, AUC, and maximum concentration were summarized by wave, microenvironment, and context as well.

To understand the impact of time-activity patterns on PM_2.5_ primary combustion peak exposure, we first aggregated the minute-level data into hourly data. For each participant-day, we summarized the time (in minutes) that the participant was awake in the hour (general sleep and wake-up time were collected at enrollment, and more resolved sleep and wake-up time during each day of monitoring were collected using EMA surveys), time (in minutes) spent in each context or microenvironment in the hour, and number, total AUC and total duration for peaks identified in the hour. For peaks occurring across hours, we counted them in the hour when the peak started.

Then, a total of three generalized linear mixed-effects models (GLMMs) with random intercepts for each participant were built to model three characteristics of PM_2.5_ peaks at the person-hour level: total AUC, the total duration of time, and the number of peaks. Negative binomial family functions were fitted in GLMMs because the three outcomes had over-dispersed distributions. Additionally, a zero-inflated portion was added to each model due to the presence of excessive zero values in the three dependent variables. Each of the three GLMMs had the same covariates of interest, including the study wave (1st trimester, 3rd trimester [reference group], and 4–6 months postpartum), weekends vs. weekdays (reference group), minutes of wake-up time in an hour), staying indoors (any hour with more than 10 min spent indoors were characterized as an indoor hour) vs. outdoors (reference group) and time spent in each of the nine microenvironments in an hour. Because personal PM_2.5_ exposure might be indirectly affected by meteorological factors (e.g. dictating ventilation patterns of the home), we further adjusted for ambient daily average temperature for participants at their home residential locations (obtained from the gridmet 4×4 km model (Abatzoglou, 2013)). Covariates were kept the same for the continuous and zero-inflated parts of each GLMM.

### 2.7. Sensitivity analyses

Sensitivity analysis was conducted to assess the impact of monitoring compliance on the temporal and spatial patterns of PM_2.5_ primary combustion peak exposures. The compliance information was first merged with above hourly level data. Then, we ran the same GLMMs with further adjustment for wearing compliance (*i.e*., a three-level categorical variable of optimal [reference group], sub-optimal and worst compliance) in the continuous and zero-inflated parts.

Peak identification was conducted in SAS v9.4 (SAS Institute, Inc., Cary, NC, USA.) where the SAS code can be find in a previous publication (Croghan and Williams, 2006). All the other analyses were conducted using R 4.0.2 (R Foundation for Statistical Computing, Vienna, Austria). The glmmTMB package (version 1.0.2.1) in R was used for GLMMs. Exponentiated effect estimates (on a multiplicative scale) and odds ratios were reported for the continuous and zero-inflated part of GLMMs, respectively.

## 3. Results

### 3.1. Descriptive statistics

A total of 63 out of 65 participants enrolled in the study had completed at least 6 hours per day of personal monitoring and were included in the final data analysis. Out of the 63 participants completed the monitoring in their 1^st^ trimester (with mean [SD] of gestational age of 15.5 [3.2] weeks), 52 of them completed the monitoring in their 3^rd^ trimester (with mean [SD] of gestational age of 30.0 [1.9] weeks) and 51 of them completed the last wave of monitoring during 4–6 months postpartum. The reasons for not completing all the three waves monitoring included withdrawal from the study, time conflicts, baby born before the 3^rd^ trimester wave assessment, could not wear equipment at work, and moved to another state. From the 63 participants, a total of 618 person-days of monitoring data with 888,361 min of PM_2.5_ mass concentrations were collected.

All participants were Hispanic women, aged 28.7 years on average at enrollment. About 54 % of them were born outside of the U.S. and had lived in the U.S. for an average of 14.2 years. In general, the study participants had relatively low household income and education levels, already had one or more children, and met criteria for obesity or over-weight before pregnancy (Table 1).

Participants were exposed to PM_2.5_ concentrations with GM of 19.2 mg/m^3^ (GSD = 2.0). Comparing between different stages of pregnancy and early postpartum, participants tended to be exposed to higher GM and GSD of PM_2.5_ after the birth of their children. Participants were exposed to higher PM_2.5_ concentrations at industrial locations (*e.g*., warehouses, auto repair shops) and transportation locations (*e.g*., Los Angeles Union Station); while their exposure levels during vehicular trips were the lowest compared to other contexts. Also, participants were more likely to be exposed to higher concentrations while outdoors, but more variable PM_2.5_ exposure in indoor microenvironments (Table 2).

**Fig. 2.**
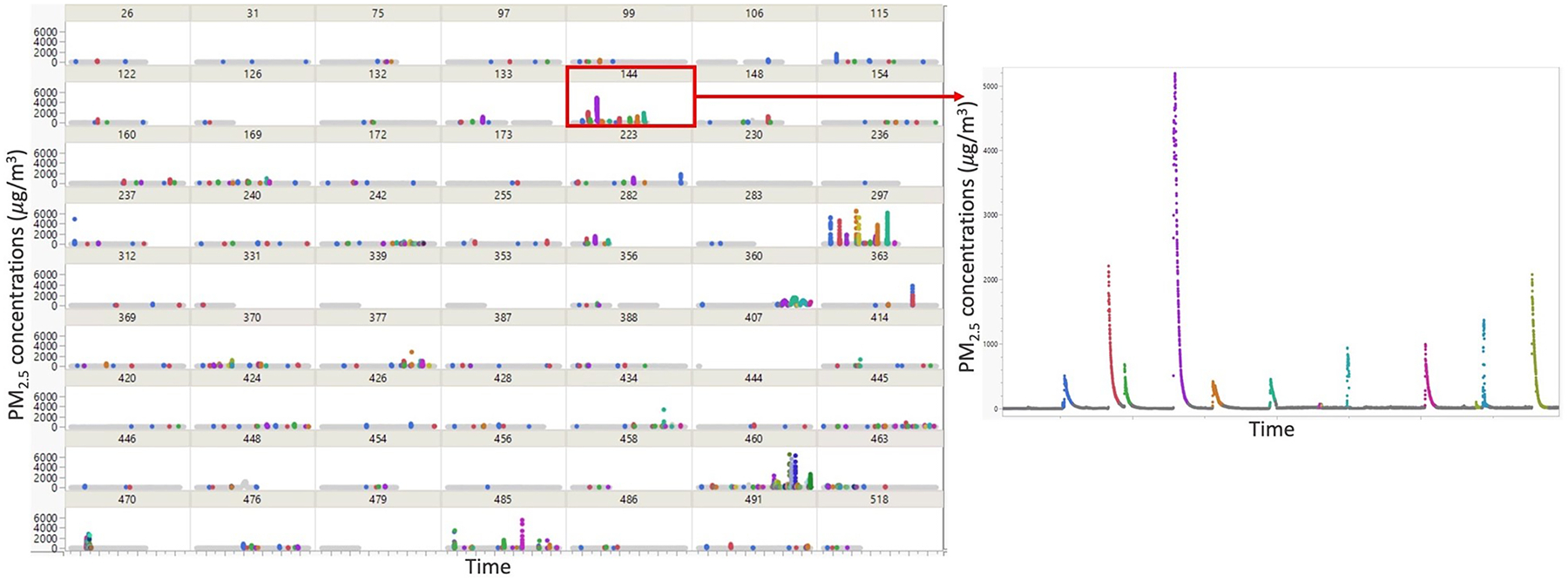
Identified peaks from the time-series PM_2.5_ concentration data (all the identified primary combustion peaks are color coded in the figure).

With the highly time and space resolved personal PM_2.5_ exposure data, we identified a total of 490 PM_2.5_ exposure peaks that were likely from primary combustion sources (Fig. 2), of which 95 peaks had unclassified microenvironment and context and were excluded from the analyses. The total 395 peaks with identified microenvironment and context lasted for 3.3 % of the total personal sampling time. No peak was identified at transportation locations likely because of the limited number of stays detected at transportation locations (total sampling time in the sub-group was the shortest at 202 mins). Peaks occurring 4–6 months postpartum, generally, had longer durations, larger AUCs, and higher maximum concentrations as compared to peaks in pregnancy. Comparing between different contexts, peaks at industrial locations tended to last longer, peaks at home residential locations had larger AUCs, and peaks at facility, office, and education locations had the highest maximum concentrations. Primary combustion peaks lasted slightly longer indoors and had larger AUCs and maximum concentrations as compared to outdoor peaks (Table 3).

### 3.2. The impact of time-activity on primary combustion related PM_2.5_ peak exposures

**Fig. 3.**
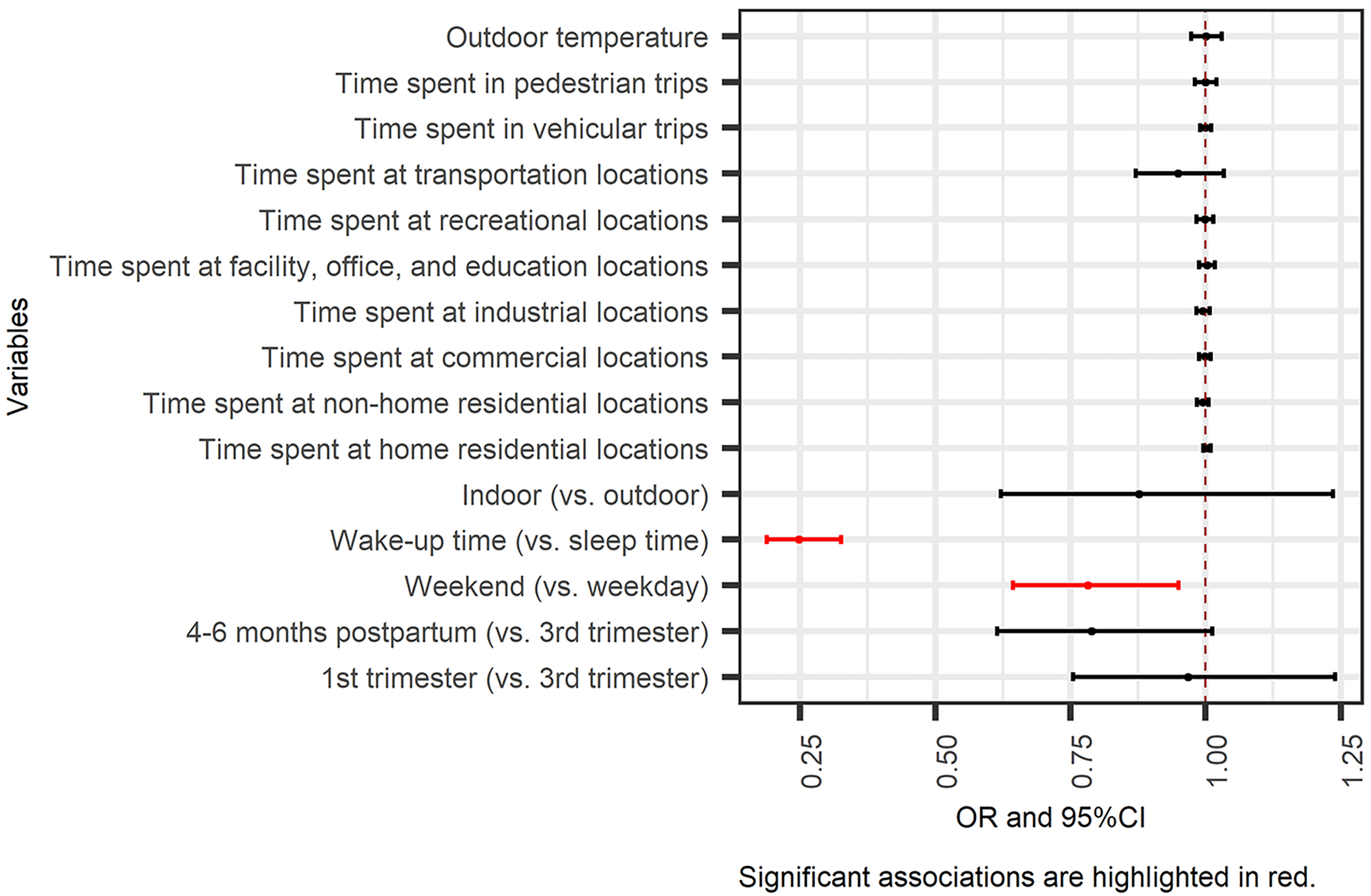
Associations of context/microenvironment and time periods with the odds of PM_2.5_ peaks from primary combustion (the effect is for each minute change in time spent at different contexts or trips and each°C degree change in temperature).

**Fig. 4.**
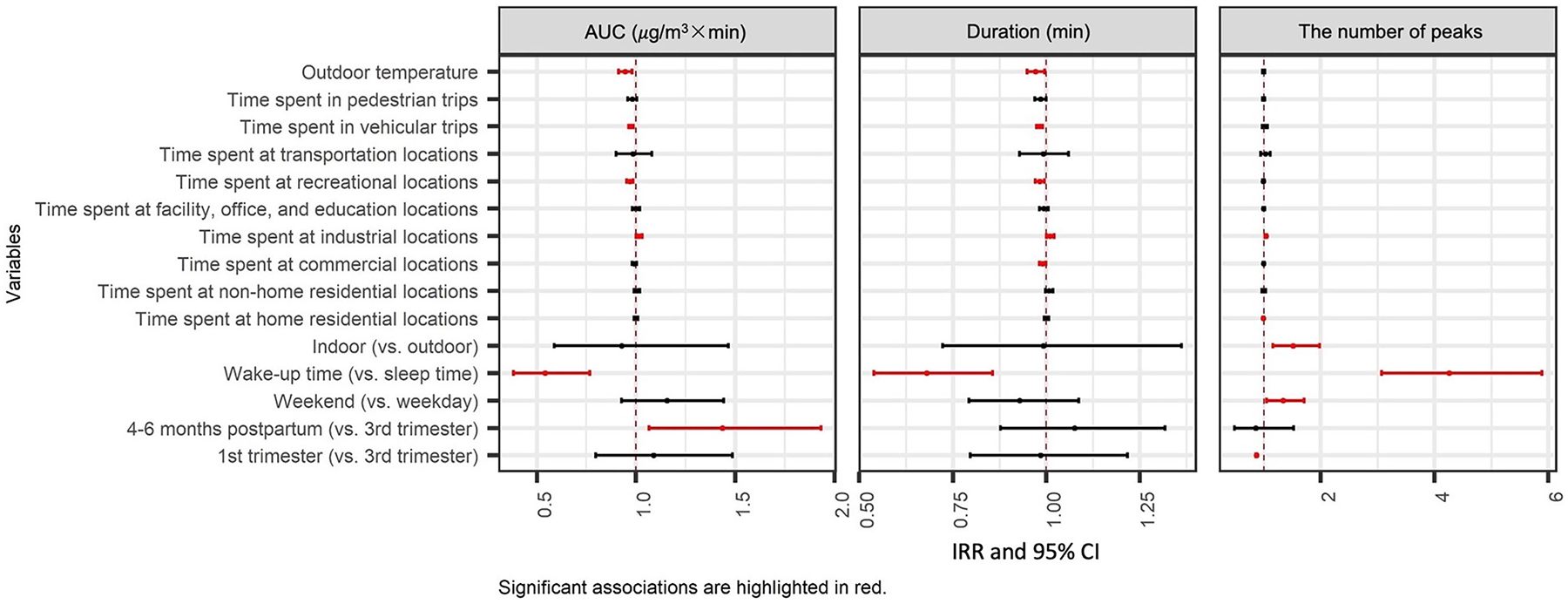
Associations of context/microenvironment and time periods with the magnitude of PM_2.5_ peaks from primary combustion (the effect is for each minute change in time spent at different contexts or trips and each°C degree change in temperature).

Results of the zero-inflated GLMMs are summarized in Figs. 3 and 4 (detailed estimates in Tables A1 and S2). All the significant results at p-value < 0.05 are highlighted in red.

The zero-inflated part of GLMMs indicated how the spatial and temporal variables impacted the odds of experiencing PM_2.5_ peaks in an hour. Results are presented as odds ratios (ORs) with 95 % confidence intervals (CIs). The odds of being exposed to PM_2.5_ peaks were 21.8 % lower (OR = 0.782, 95 % CI: 0.644 – 0.950) on weekends and 75.3 % lower (OR = 0.247, 95 % CI: 0.188 – 0.325) during wake-up time, as compared to weekdays and sleep time, respectively (Fig. 3).

The continuous part of GLMMs compared hours with at least one peak and indicated the associations between spatial and temporal variables with the magnitude of peak exposures defined in several ways. The primary metric used in the analysis was the AUC of a peak, which represented the potential PM_2.5_ dose a participant might be inhaling when exposed to a peak (integration of concentration over time). Other metrics used in the analysis include the duration (in mins) and number of peaks exposed in an hour. Results of the continuous part of GLMMs are presented as incidence rate ratios (IRRs) with 95 % CIs.

During early pregnancy, participants were exposed to a smaller number of peaks than late pregnancy (0.868, 95 % CI: 0.857–0.878); the AUC and duration of peak exposures had no difference by stage of pregnancy. However, after delivery, participants were more likely to be exposed to larger peaks (*i.e*., larger AUC [1.436, 95 % CI: 1.066–1.934]), but similar duration and number of peaks as compared to exposures during the 3rd trimester. The participants were exposed to more peaks with similar duration and size on weekends (1.334, 95 % CI: 1.045–1.522) as compared to weekdays across all periods. Comparing wake-up time and sleep time, participants exposed to more peaks (4.250, 95 %CI: 3.070–5.884) but of smaller size (0.542, 95 % CI: 0.383–0.767) and shorter duration (0.679, 95 % CI: 0.539–0.857) when they are awake.

Comparing different microenvironments, participants were exposed to a larger number of peaks (1.514, 95 % CI: 1.157 – 1.981) with similar size and duration when they stayed indoors (at home or other indoor locations) compared to outdoors.

Comparing different contexts, participants were exposed to a slightly smaller number of peaks (0.989, 95 % CI: 0.984–0.995) when they spent more time at home. The duration of peaks decreased by 1 % (0.990, 95 % CI: 0.982–0.998) when they spent one more minute at commercial locations. When spending an additional minute at an industrial location, participants were exposed to larger (1.016, 95 % CI: 1.001–1.032), longer (1.010, 95 % CI: 1.000–1.021) and a greater number (1.036, 95 % CI: 1.020–1.052) of peaks. When participants spent one more minute at recreational location or in vehicular trips, peaks they were exposed to were smaller in size (recreational locations: 0.969, 95 % CI: 0.954–0.985; vehicular trips: 0.975, 95 % CI: 0.963–0.988) and shorter in duration (recreational locations: 0.983, 95 % CI: 0.971–0.995; vehicular trips: 0.982, 95 % CI: 0.974 – 0.990); whereas the number of peaks they were exposed to stayed unchanged.

### 3.3. Monitoring compliance and sensitivity analyses

**Fig. 5.**
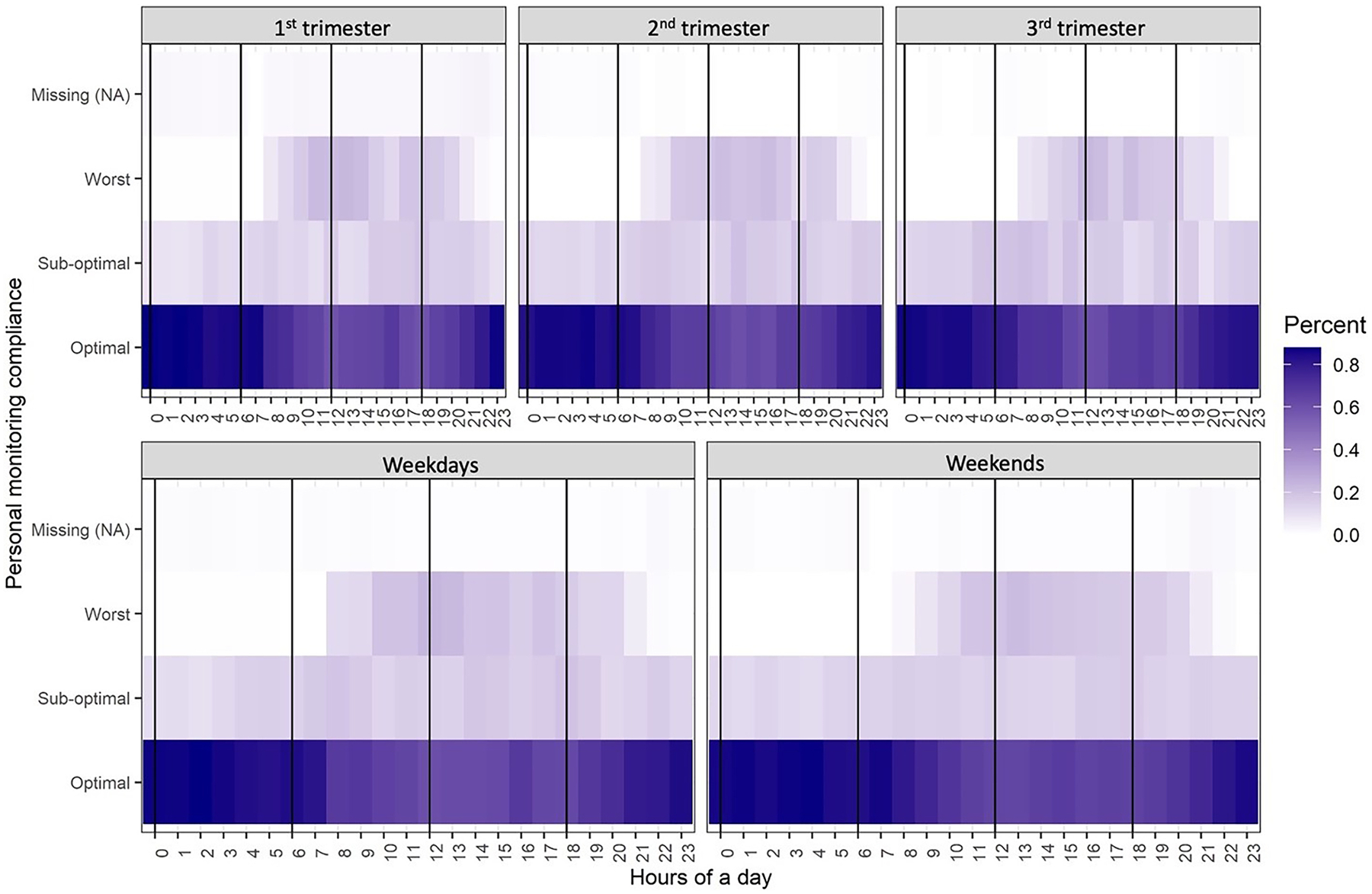
Summary of personal monitoring compliance by waves and weekdays/weekends.

Fig. 5 shows the percentage of minute-level data in each of the categories of monitoring compliance (*i.e*., optimal, sub-optimal, worst, and missing) by hour of day. Darker cells in Fig. 5 represent larger percent of data in the corresponding category. The general pattern of monitoring compliance was similar across study waves. Specifically, participants had the best monitoring compliance during late night and early morning, and the worst compliance during daytime (8 am–8 pm). However, the compliance data was relatively complete with very little missingness. Comparing wear compliance by weekdays and weekends, the decline in compliance on weekends occurred later in the morning (around 10 am)

**Fig. 6.**
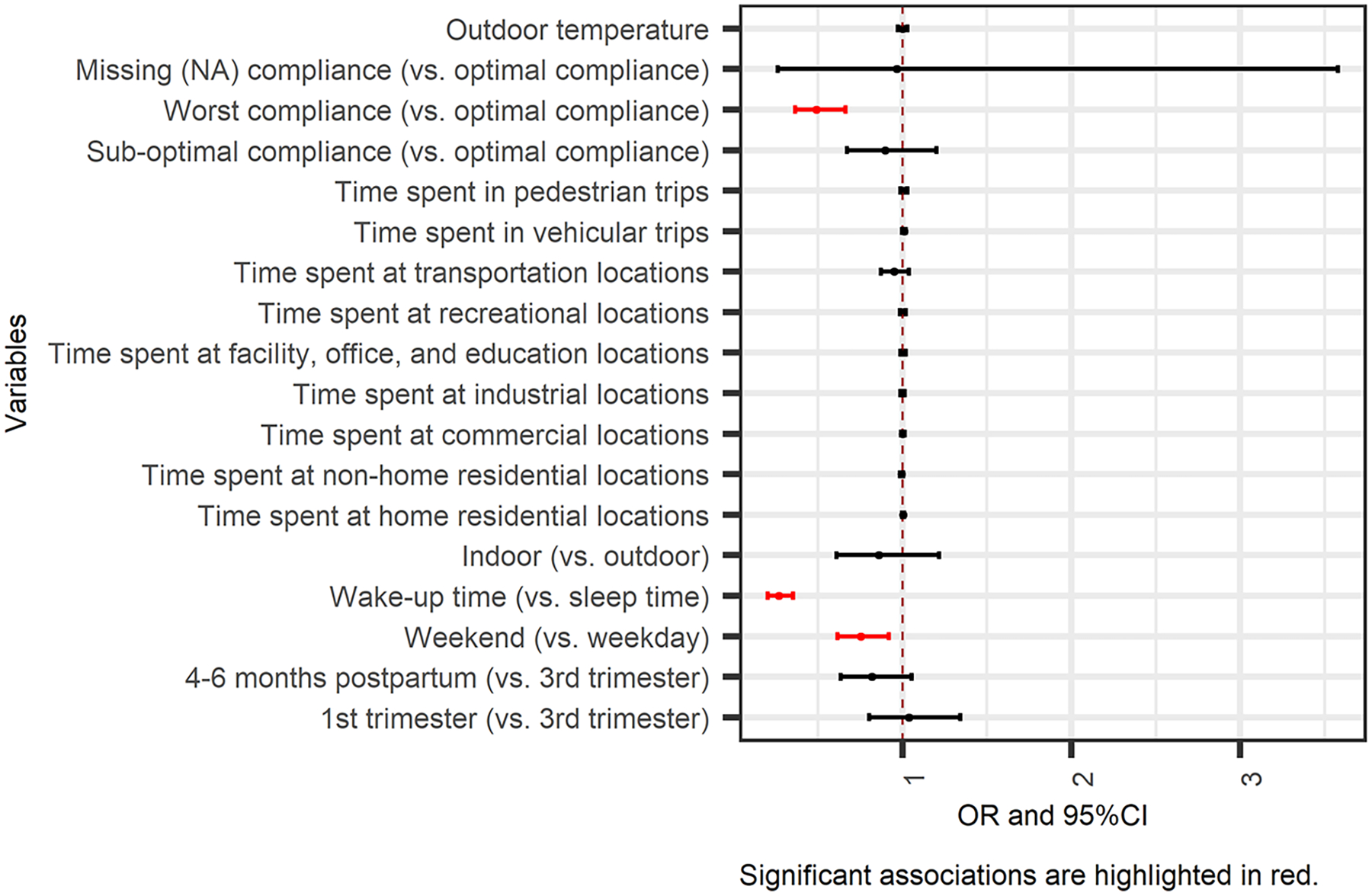
Associations of context/microenvironment and time periods with the odds of PM_2.5_ peaks from primary combustion after adjusting for monitoring compliance (the effect is for each minute change in time spent at different contexts or trips and each°C degree change in temperature).

**Fig. 7.**
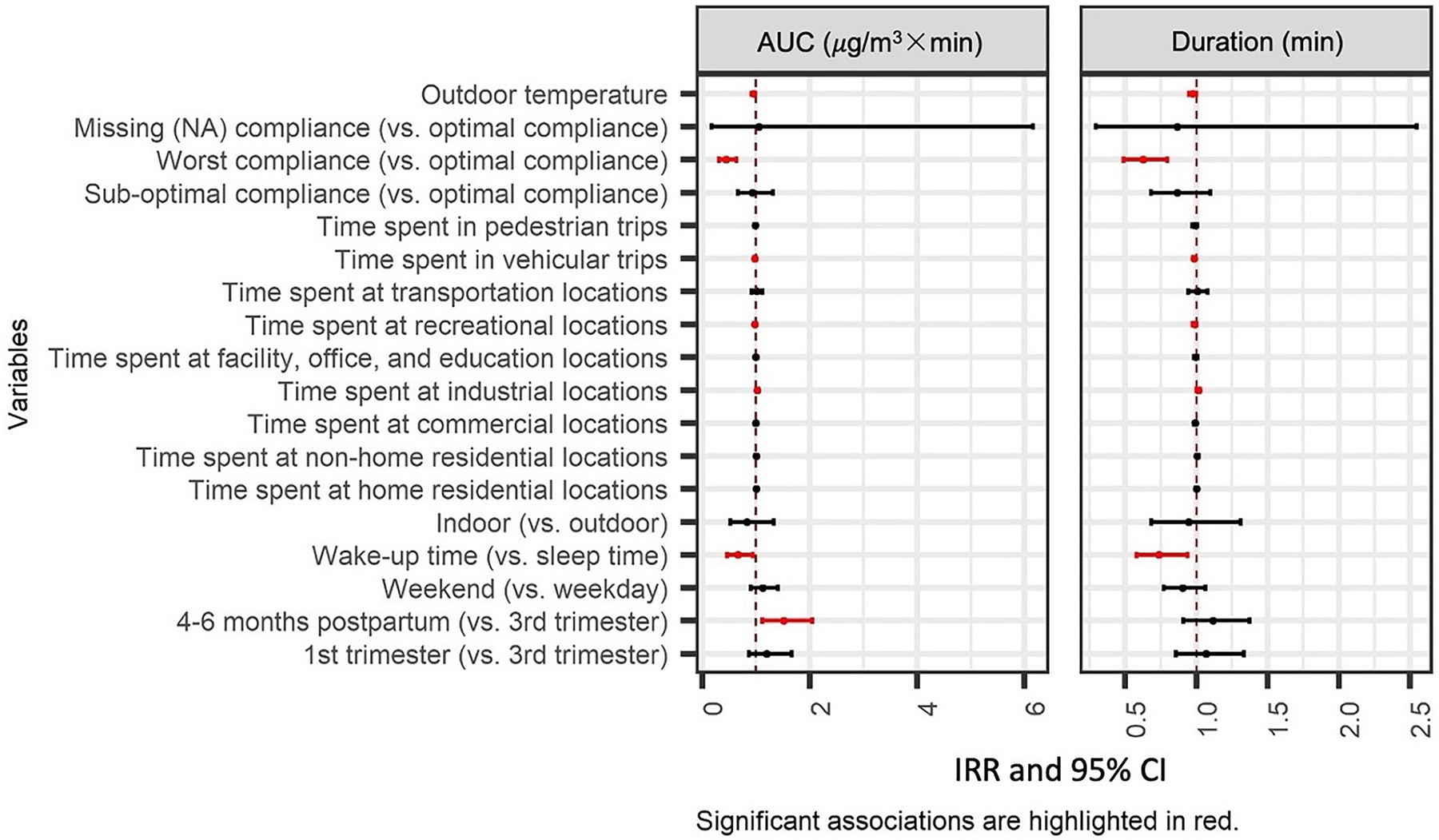
Associations of context/microenvironment and time periods with the magnitude of PM_2.5_ peaks from primary combustion after adjusting for monitoring compliance (the effect is for each minute change in time spent at different contexts or trips and each°C degree change in temperature).

In the sensitivity analyses, we further adjusted for monitoring compliance in the GLMM. While the model for the number of peaks did not converge (possibly due to the relatively large correlations between the number of peaks detected and monitoring compliance), the GLMM results for AUC and duration of peaks are summarized in Figs. 6 and 7 (details shown in Table A3 and A4). After adjusting for compliance, the results remained similar to the main models with no compliance adjustment. Interestingly, the worst category of monitoring compliance was negatively associated with AUC and duration of peaks in both the zero-inflated and continuous part of the models as compared to times with optimal compliance.

## 4. Discussion

This is a panel study using innovative, personal data capture strategies, including wearables and sensors and real-time self-report, to characterize personal PM_2.5_ exposure, identify PM_2.5_ peaks exposure from primary combustion sources, and assess the impact of time-activity patterns in different contexts and microenvironments on PM_2.5_ peaks exposure across pregnancy and early postpartum. In this work, we found that participants were exposed to higher PM_2.5_ concentrations after their children were born, when they stayed at industrial locations, and when they were indoors as compared to exposure levels during pregnancy and at other microenvironments or outdoor locations. We also successfully identified 490 PM_2.5_ exposure peaks that were likely from primary combustion sources based on the continuous personal total PM_2.5_ measurements. Combined with continuously tracked GPS locations, we found consistent associations between time-activity patterns and contexts and primary combustion peak exposures. That is, pregnant women were more likely to be exposed to longer and larger primary combustion PM_2.5_ peak during sleep time and when they stayed at industrial locations; however, spending more time at parks and open spaces were associated with shorter and smaller PM_2.5_ peak exposures.

Based on the continuous part of GLMMs, we found robust associations between more time spent at parks and open spaces and in vehicular trips with exposure to shorter and smaller peaks. The reason for experiencing smaller and briefer peaks of exposure when spending more time in vehicular trips could be attributed to the ventilation system of the private or public transit vehicles. This phenomenon is particularly prevalent in Los Angeles, in which the hot weather prompts use of air conditioning in personal vehicles and public transit (buses and trains). The enhanced filtration, dilution and mixing within an air-conditioned cabin can result in a cleaner microenvironment, reducing the likelihood of exposure to primary combustion peaks. Although vehicular trips are not encouraged because of the air pollution emissions and reduced physical activity level, the results potentially suggest that improving in-vehicle ventilation can reduce peak PM_2.5_ exposure although further research is needed. For the decreased peak exposure at parks and open spaces, although previous studies did not investigate the distribution of peaks exposure, an increasing amount of evidence suggested lower personal PM_2.5_ exposures at greenspace including in our work (Xu et al., 2022, Mueller et al., 2022). Possible mechanisms for the negative associations include lower density of pollution sources with greenspace, removal of particles by vegetation, or facilitating the dispersion of pollutants in open areas (Salmond et al., 2016, Xing and Brimblecombe, 2020).

We also found associations of more time spent at industrial locations with exposures to a larger number and longer and larger (*i.e*., increased AUC) primary combustion peaks. The positive associations between time spent at industrial locations and peak exposure could be explained by industrial combustion processes or related traffic. While many of the industrial locations visited by participants in the study were mixed land use locations (especially in downtown LA, *e.g*., warehouses behind a residential building with restaurants and auto repairs nearby), and it is challenging to distinguish which of these uses the participant visited or experienced, further studies are warranted to confirm if the increased peak exposure are attributed to industrial locations or the mixed land use context of these stays.

We found participants were more likely to be exposed to primary combustion peaks during weekdays and while asleep as compared to weekends and while awake. According to previous literature, less primary combustion peak exposure on weekends may be related with more visits to parks and open space on weekends and spending less time in trips (exposed less to traffic emissions) (Yi et al., 2022) or changes in routine activities or behaviors that emit PM_2.5_ from combustion on weekdays (e.g., less cooking on weekends). Less peak exposure during wake-up time might be related with being at work locations that may have better filtration (*e.g*., those with air conditioning) or fewer sources than being at home. It may also be related to the observed slightly lower compliance rate during daytime. Participants might be more likely to opt to place the monitor next to them as instructed than carry it during these hours, so that the measured exposure did not represent their actual activities. While humidity can impact the reading of PM_2.5_ sensors, we have checked that the relative humidity data of PM_2.5_ sensors were not significantly different between day-time and night-time.

We also compared total personal PM_2.5_ exposures at different stages of pregnancy and postpartum and found higher personal PM_2.5_ exposure after the birth of babies than during pregnancy. Our study is one of very few studies that measured personal PM_2.5_ exposure over time during different stages of pregnancy or postpartum. Most previous studies estimated postnatal PM_2.5_ exposure for newborn babies at participants’ residence to estimate the health impact of early life air pollution exposure on children’s health in later life, (Goyal et al., 2019, McGuinn et al., 2022, Vanoli et al., 2022, Chang et al., 2022) very little is known about the personal total PM_2.5_ exposure for pregnant women during the postpartum period. A study followed 524 pregnant women living in rural areas of southwest China to repeatedly measure their personal PM_2.5_ exposure and found slightly lower but insignificant PM_2.5_ exposure changes during the 3rd trimester compared to the 1^st^ trimester. Our study, though among Hispanic women living in urban areas in the US showed similar result that there were no differences in personal PM_2.5_ exposure between late and early pregnancy. The higher PM_2.5_ exposure levels at 4–6 months postpartum compared to during pregnancy found in this study may be explained by differences in time-activity patterns or behaviors in the home before and after delivery, as shown in our previous studies among the same participants (Yi et al., 2022). For example, participants spent more time at home and non-home residential locations, but less time at parks and open spaces after the birth of their children as compared to pregnancy. It is also possible cooking, cleaning, or other household activities might change or increase in the postpartum period (Gjerdingen and Chaloner, 1994, Gjerdingen and Center, 2005).

Participants in this study showed good compliance with the personal monitoring across study waves (*i.e*., different stages of pregnancy and postpartum) and weekdays and weekends. Compliance is one of the major challenges in environmental studies that involve personal monitoring, and this is likely even more of a challenge in pregnancy studies. Our study found poorer compliance was negatively associated with PM_2.5_ peak exposures. This finding indicated the importance of personal monitoring, as its ability to capture PM_2.5_ exposure not only from outdoor and indoor sources, but also “personal cloud” (localized generation of particulate matter due to human activities) (Brown et al., 2009). Our study also shows the feasibility of personal monitoring among pregnant women if the monitoring protocol is carefully designed. Personal monitoring can be burdensome, especially for the pregnant population. We programmed all the exposure devices to turn on and off automatically, without the need to be charged or manipulated. These design aspects along with carefully crafted instructions help reduce the burden of personal monitoring and improve monitoring compliance. We noticed that personal monitoring compliance declined during daytime (mean = 66.5 % with range between 58.6 % and 100 %). However, a previous study had found that as long as the compliance reached 40 %, the personal monitoring data would still be usable and would not misrepresent exposures measured longitudinally (Lawless et al., 2012).

To our knowledge, this study is the first of its kind to investigate the distribution of personal PM_2.5_ peak exposures that are highly likely from primary combustion processes. From the perspective of exposure science, this study used data driven detection algorithms to identify these source-specific peaks to help exposure scientists understand where and when a study participant is exposed to primary combustion PM_2.5_—information that cannot be extracted from integrated filter based PM_2.5_ samples. Previous studies including from our team relied on filter based integrated PM_2.5_ samples to analyze chemical components and apportion sources which is also very valuable and may detect sources that do not have a characteristic “shape” (Xu et al., 2024). From the perspective of environmental health, identifying PM_2.5_ primary combustion peaks provides the opportunity to explore alternative exposure metrics and to understand their health effects and design intervention or exposure mitigation strategies. It also provides the opportunity for epidemiological studies to examine if certain health outcomes are triggered by peak exposures instead of the commonly used average level of air pollution during time windows of interests. In addition, identifying PM_2.5_ primary combustion peaks in epidemiological studies could allow investigations into the potential differential toxicity of combustion vs non-combustion related PM_2.5_ sources. From the perspective of health interventions, the identified associations between different contexts/microenvironments and PM_2.5_ peak exposure inform strategies to reduce prenatal and postnatal PM_2.5_ exposures in key areas or activities. For example, our study found spending more time at parks and open space was helpful for avoiding exposure to PM_2.5_ peaks.

This study also has some limitations. First, we only collected personal exposure data from 63 women in Los Angeles, CA, as part of a nested study within a larger cohort study. Although the study results may not be generalized to other populations, this study provide important evidence for a vulnerable population. While this sample size is small for population-level studies that look at between-person differences in exposure, it is actually reasonable for personal exposure monitoring studies, especially with the repeated measurements from the same participants over time and over 14,500 person-hours of data. Second, despite the overall high level of compliance across the study, we found slightly lower monitoring compliance during wake-up time. However, wear compliance was still satisfactory (participants wore sensors or had sensors in the same room for a mean of 82.5 % of the sampling time during awaken time) to help understand personal PM_2.5_ exposures and did not impact main findings of this study (as shown in the sensitivity analyses). Finally, our study cannot verify whether identified PM_2.5_ peaks were truly from primary combustion processes or which specific ones (*e.g*. burning a candle or cooking etc.) as we identified these based on PM_2.5_ mass concentration signals with no information on chemical composition or other annotation. It is still a challenge with current sampling technologies to collect highly time-resolved and chemically speciated PM_2.5_ samples that would enable these kinds of studies in the future and provide more comprehensive evidence on the multiple, complex sources impacting personal PM_2.5_ exposures.

## 5. Conclusion

This study continuously monitored the personal PM_2.5_ exposure for women during their pregnancy and early postpartum, which are critical exposure windows for women and children’s health. Our study shows the feasibility of using statistical methods to identify PM_2.5_ peak exposure that are highly characteristic of primary combustion processes. Combined with time-activity, context, and mobility data, this study further investigated the spatial and temporal determinants of PM_2.5_ peak exposures. Results of this study shed light on potential intervention strategies to minimize prenatal and postnatal personal PM_2.5_ exposures, including encouraging visits to parks and open spaces and improving indoor air quality when indoors. Methods used in the current study can also be applied to future epidemiological studies to further explore the health effects of PM_2.5_ peak exposure from primary combustion sources.

## Supplementary Material

1

## Figures and Tables

**Table 1:** Descriptive statistics of participant characteristics.

Characteristics^#^	US-born	Foreign-born	Total
**N**	29 (46.0 %)	34 (54.0 %)	63 (100.0 %)
**Age (years)**	26.4 (4.7)	30.6 (6.3)	28.7 (6.0)
**Household size (number of people living in the same home)**	5.3 (2.2)	5.0 (1.9)	5.1 (2.0)
**Years living in the U.S. (years)**	NA	14.2 (8.6)	NA
**Marital status**			
Married	10 (34.5 %)	13 (38.2 %)	23 (36.5 %)
Living together	13 (44.8 %)	15 (44.1 %)	28 (44.4 %)
Divorced or separated	0 (0.0 %)	1 (2.9 %)	1 (1.6 %)
Single (never married)	5 (17.2 %)	4 (11.8 %)	9 (14.3 %)
Decline to answer	1 (3.4 %)	1 (2.9 %)	2 (3.2 %)
**Household income per year**			
< $30,000	17 (58.6 %)	17 (50.0 %)	34 (54.0 %)
^3^ $30,000	7 (24.1 %)	7 (20.6 %)	14 (22.2 %)
Do not know	5 (17.2 %)	10 (29.4 %)	15 (23.8 %)
**Education**			
< High school	4 (13.8 %)	15 (44.1 %)	19 (30.2 %)
^3^ High school	25 (86.2 %)	19 (55.9 %)	44 (69.8 %)
**Maternal parity**			
First-born	11 (37.9 %)	5 (14.7 %)	16 (25.4 %)
Second born or greater	18 (62.1 %)	29 (85.3 %)	47(74.6 %)
**Pre-Pregnancy BMI**			
Normal weight (18.5–24.9 kg/m^2^)	9 (31.0 %)	8 (23.5 %)	17 (27.0 %)
Overweight (25.0–29.9 kg/m^2^)	10 (34.5 %)	13 (38.2 %)	23 (36.5 %)
Obesity (^3^ 30.0 kg/m^2^)	10 (34.5 %)	13 (38.2 %)	23 (36.5 %)

# Continuous variables are presented as mean (SD) and categorical variables are summarized as number (percentage).

**Table 2:** Minute-level personal PM_2.5_ exposures by wave, context, and microenvironment.

PM2.5 mass concentrations (mg/m^3^)	Median	GM	GSD	*p*-value*
**Total**	11.8	11.6	2.0	
**Wave**				<0.001
1st trimester	11.7	11.5	1.9	
3rd trimester	11.8	11.3	2.0	
4–6 months postpartum	11.5	12.1	2.2	
**Context of Stay Locations** ^#^				<0.001
Home residential location	11.8	11.7	2.0	
Non-home residential location	11.6	11.2	1.8	
Commercial location	11.0	9.8	2.4	
Industrial location	13.4	13.9	2.3	
Facility, office, and education location	11.1	10.5	2.0	
Recreational location	11.5	10.9	1.7	
Transportation location	12.5	14.2	1.7	
**Trips by Mode**				<0.001
Pedestrian trip	12.0	11.4	2.0	
Vehicular trip	10.8	10.2	2.0	
**Microenvironment** ^#^				<0.001
Indoor	11.7	11.5	2.0	
Outdoor	12.1	11.9	1.7	

# Personal PM_2.5_ concentrations were summarized across all occurrences of a particular microenvironment or context.

* *p*-value was based on Kruskal-Wallis rank-sum test. Pairwise comparisons between groups (Wilcoxon rank-sum test with Benjamini & Hochberg method to adjust *p*-values for multiple comparisons) were all significant, except for the comparison between industrial location and transportation location.

**Table 3:** Summary of primary combustion peaks by wave, context, and microenvironment.

Primary combustion peaks	Number of peaks	Total duration of peaks (min)	Percent of sampling time^#^	GM of duration (min)	GSD of duration	GM of AUC (μg/m^3^×min)	GSD of AUC	GM of maximum concentration (μg/m^3^)	GSD of maximum concentration
**Total Waves**	395	18,404	3.3 %	28.1	2.7	2036.5	4.2	139.0	2.7
1^st^ trimester	137	6,219	1.9 %	26.9	2.8	1973.1	4.0	139.2	2.4
3^rd^ trimester	119	4,777	1.8 %	28.8	2.3	1919.1	3.6	130.5	2.8
4–6 months postpartum	140	7,416	2.7 %	28.4	3.0	2184.7	5.0	146.0	2.9
**Context of Stay Locations**									
Home residential location	289	14,747	2.5 %	32.0	2.7	2423.7	4.3	149.4	2.7
Non-home residential location	18	983	4.3 %	34.6	2.6	1869.3	3.3	104.1	2.3
Commercial location	27	627	2.0 %	16.5	2.2	1183.2	3.5	119.6	2.9
Industrial location	18	1,060	9.7 %	27.0	3.4	1801.5	4.3	107.5	2.0
Facility, office, and education location	9	228	1.6 %	18.3	2.3	1633.1	4.5	154.3	4.0
Recreational location	6	66	1.4 %	9.1	1.8	447.0	2.7	78.4	1.7
Transportation location	0	NA	NA	NA	NA	NA	NA	NA	NA
**Trips by Mode**									
Pedestrian trip	9	178	2.1 %	17.1	1.8	1117.5	2.8	122.9	2.7
Vehicular trip	19	515	1.4 %	16.0	2.5	904.7	3.6	117.2	2.9
**Microenvironment**									
Indoor	307	15,372	2.8 %	30.6	2.7	2319.8	4.3	148.0	2.8
Outdoor	57	2,276	2.0 %	23.8	2.7	1497.1	3.6	108.5	2.2

# The percent of sampling time = total duration of peaks / total sampling time in the sub-group ´100 %

## Data Availability

The data that has been used is confidential.
