# Supplementary material for: Spatial and temporal determinants of particulate matter peak exposures during pregnancy and early postpartum
Source: Environ Adv. Author manuscript; Available in PMC 2024 Nov 21. (PMC11580741; doi:10.1016/j.envadv.2024.100557)
Supplement: 1 [file NIHMS2032471-supplement-1.docx]

Spatial and Temporal Determinants of Particulate Matter Peak Exposures during Pregnancy and Early Postpartum

Yisi Liu^1,2^*, Li Yi^3^, Yan Xu^1^, Jane Cabison^1^, Sandrah P. Eckel^1^, Tyler B. Mason^1^, Daniel Chu^1^, Nathana Lurvey^4^, Deborah Lerner^4^, Jill Johnston^1^, Theresa M. Bastain^1^, Shohreh F. Farzan^1^, Carrie V. Breton^1^, Genevieve F. Dunton^1^, Rima Habre^1,5^

1 Department of Population and Public Health Sciences, Keck School of Medicine, University of Southern California, Los Angeles, CA, USA

2 Department of Epidemiology and Environmental Health, College of Public Health, University of Kentucky, Lexington, KY, USA

3 Harvard T.H. Chan School of Public Health, Boston, MA, USA

4 Eisner Health, Los Angeles, CA, USA

5 Spatial Sciences Institute, University of Southern California, Los Angeles, CA, USA

* Corresponding author at: Department of Epidemiology and Environmental Health, College of Public Health, University of Kentucky, 111 Washington Ave, Room 214B, Lexington, KY 40536, USA. *E-mail address:* yisi.liu@uky.edu (Y. Liu)

**Table of Content**

**Table A1** Associations of context/microenvironment and time periods with the odds of PM_2.5_ peaks from primary combustion (zero-inflated part of GLMMs).

**Table A2**. Associations of context/microenvironment and time periods with the magnitude of PM_2.5_ peaks from primary combustion (continuous part of GLMMs).

**Table A3**. Sensitivity analyses results for the associations of context/microenvironment and time periods with the odds of PM_2.5_ peaks from primary combustion (zero-inflated part of GLMMs).

**Table A4**. Sensitivity analyses results for the associations of context/microenvironment and time periods with the magnitude of PM_2.5_ peaks from primary combustion (continuous part of GLMMs).

**Table A1**. Associations of context/microenvironment and time periods with the odds of PM_2.5_ peaks from primary combustion (zero-inflated part of GLMMs, the effect is for each minute change in time spent at different contexts or trips and each °C degree change in temperature).

| **Variables** | **OR** | **95% CIs** | |
| --- | --- | --- | --- |
| 1st trimester (vs. 3rd trimester) | 0.967 | 0.755 | 1.240 |
| 4-6 months postpartum (vs. 3rd trimester) | 0.789 | 0.615 | 1.012 |
| Weekend (vs. weekday) | **0.782** | **0.644** | **0.950** |
| Wake-up time (vs. sleep time) | **0.247** | **0.188** | **0.325** |
| Indoor (vs. outdoor) | 0.876 | 0.622 | 1.236 |
| Time spent at home residential locations | 1.003 | 0.996 | 1.010 |
| Time spent at non-home residential locations | 0.995 | 0.984 | 1.005 |
| Time spent at commercial locations | 0.999 | 0.988 | 1.009 |
| Time spent at industrial locations | 0.995 | 0.983 | 1.008 |
| Time spent at facility, office, and education locations | 1.002 | 0.988 | 1.017 |
| Time spent at recreational locations | 0.998 | 0.983 | 1.014 |
| Time spent at transportation locations | 0.949 | 0.871 | 1.034 |
| Time spent in vehicular trips | 1.000 | 0.990 | 1.010 |
| Time spent in pedestrian trips | 1.000 | 0.981 | 1.020 |
| Outdoor temperature | 1.001 | 0.973 | 1.030 |

Significant associations are highlighted in bold.

**Table A2**. Associations of context/microenvironment and time periods with the magnitude of PM_2.5_ peaks from primary combustion (continuous part of GLMMs, the effect is for each minute change in time spent at different contexts or trips and each °C degree change in temperature).

| **Variables** | **AUC (𝜇g/m^3^×min)** | | | **Duration (min)** | | | **Peak** **number** | | |
| --- | --- | --- | --- | --- | --- | --- | --- | --- | --- |
|  | Point estimates | 95% CIs | | Point estimates | 95% CIs | | Point estimates | 95% CIs | |
| 1st trimester (vs. 3rd trimester) | 1.088 | 0.797 | 1.487 | 0.984 | 0.797 | 1.216 | **0.868** | **0.857** | **0.878** |
| 4-6 months postpartum (vs. 3rd trimester) | **1.436** | **1.066** | **1.934** | 1.075 | 0.878 | 1.317 | 0.856 | 0.482 | 1.522 |
| Weekend (vs. weekday) | 1.158 | 0.929 | 1.443 | 0.928 | 0.793 | 1.087 | **1.334** | **1.045** | **1.703** |
| Wake-up time (vs. sleep time) | **0.542** | **0.383** | **0.767** | **0.679** | **0.539** | **0.857** | **4.250** | **3.070** | **5.884** |
| Indoor (vs. outdoor) | 0.929 | 0.589 | 1.466 | 0.992 | 0.723 | 1.362 | **1.514** | **1.157** | **1.981** |
| Time spent at home residential locations | 1.001 | 0.992 | 1.010 | 1.000 | 0.994 | 1.006 | **0.989** | **0.984** | **0.995** |
| Time spent at non-home residential locations | 1.005 | 0.990 | 1.020 | 1.007 | 0.997 | 1.018 | 0.997 | 0.960 | 1.034 |
| Time spent at commercial locations | 0.991 | 0.979 | 1.003 | **0.990** | **0.982** | **0.998** | 0.996 | 0.985 | 1.006 |
| Time spent at industrial locations | **1.016** | **1.001** | **1.032** | **1.010** | **1.000** | **1.021** | **1.036** | **1.020** | **1.052** |
| Time spent at facility, office, and education locations | 1.000 | 0.982 | 1.019 | 0.993 | 0.981 | 1.005 | 0.997 | 0.981 | 1.014 |
| Time spent at recreational locations | **0.969** | **0.954** | **0.985** | **0.983** | **0.971** | **0.995** | 0.989 | 0.973 | 1.005 |
| Time spent at transportation locations | 0.986 | 0.900 | 1.080 | 0.992 | 0.928 | 1.059 | 1.023 | 0.942 | 1.110 |
| Time spent in vehicular trips | **0.975** | **0.963** | **0.988** | **0.982** | **0.974** | **0.990** | 1.017 | 0.973 | 1.063 |
| Time spent in pedestrian trips | 0.982 | 0.959 | 1.005 | 0.985 | 0.969 | 1.000 | 0.995 | 0.978 | 1.013 |
| Outdoor temperature | **0.945** | **0.912** | **0.980** | **0.972** | **0.948** | **0.996** | 0.996 | 0.973 | 1.020 |

Significant associations are highlighted in bold.

**Table A3**. Sensitivity analyses results for the associations of context/microenvironment and time periods with the odds of PM_2.5_ peaks from primary combustion (zero-inflated part of GLMMs, the effect is for each minute change in time spent at different contexts or trips and each °C degree change in temperature).

| **Variables** | **OR** | **95% CIs** | |
| --- | --- | --- | --- |
| 1st trimester (vs. 3rd trimester) | 1.038 | 0.803 | 1.341 |
| 4-6 months postpartum (vs. 3rd trimester) | 0.817 | 0.633 | 1.055 |
| Weekend (vs. weekday) | **0.751** | **0.615** | **0.918** |
| Wake-up time (vs. sleep time) | **0.266** | **0.201** | **0.352** |
| Indoor (vs. outdoor) | 0.860 | 0.608 | 1.217 |
| Time spent at home residential locations | 1.003 | 0.996 | 1.010 |
| Time spent at non-home residential locations | 0.994 | 0.983 | 1.005 |
| Time spent at commercial locations | 1.001 | 0.990 | 1.012 |
| Time spent at industrial locations | 1.000 | 0.987 | 1.014 |
| Time spent at facility, office, and education locations | 1.003 | 0.988 | 1.018 |
| Time spent at recreational locations | 1.002 | 0.986 | 1.018 |
| Time spent at transportation locations | 0.951 | 0.872 | 1.037 |
| Time spent in vehicular trips | 1.009 | 0.998 | 1.021 |
| Time spent in pedestrian trips | 1.007 | 0.987 | 1.028 |
| Sub-optimal compliance (vs. optimal compliance) | 0.898 | 0.671 | 1.202 |
| Worst compliance (vs. optimal compliance) | **0.490** | **0.363** | **0.661** |
| Missing (NA) compliance (vs. optimal compliance) | 0.967 | 0.261 | 3.580 |
| Outdoor temperature | 1.001 | 0.972 | 1.030 |

Significant associations are highlighted in bold.

**Table A4**. Sensitivity analyses results for the associations of context/microenvironment and time periods with the magnitude of PM_2.5_ peaks from primary combustion (continuous part of GLMMs, the effect is for each minute change in time spent at different contexts or trips and each °C degree change in temperature).

| **Variables** | **AUC (𝜇g/m^3^×min)** | | | **Duration (min)** | | |
| --- | --- | --- | --- | --- | --- | --- |
|  | Point estimates | 95% CIs | | Point estimates | 95% CIs | |
| 1st trimester (vs. 3rd trimester) | 1.197 | 0.863 | 1.660 | 1.069 | 0.857 | 1.333 |
| 4-6 months postpartum (vs. 3rd trimester) | **1.512** | **1.115** | **2.051** | 1.115 | 0.906 | 1.372 |
| Weekend (vs. weekday) | 1.129 | 0.903 | 1.412 | 0.905 | 0.770 | 1.064 |
| Wake-up time (vs. sleep time) | **0.662** | **0.466** | **0.940** | **0.738** | **0.580** | **0.938** |
| Indoor (vs. outdoor) | 0.835 | 0.526 | 1.325 | 0.947 | 0.685 | 1.311 |
| Time spent at home residential locations | 1.002 | 0.993 | 1.011 | 1.001 | 0.994 | 1.007 |
| Time spent at non-home residential locations | 1.003 | 0.988 | 1.018 | 1.007 | 0.997 | 1.017 |
| Time spent at commercial locations | 0.993 | 0.982 | 1.005 | 0.992 | 0.983 | 1.000 |
| Time spent at industrial locations | **1.022** | **1.006** | **1.039** | **1.014** | **1.003** | **1.025** |
| Time spent at facility, office, and education locations | 1.001 | 0.983 | 1.020 | 0.995 | 0.983 | 1.007 |
| Time spent at recreational locations | **0.978** | **0.962** | **0.995** | **0.987** | **0.975** | **1.000** |
| Time spent at transportation locations | 1.016 | 0.927 | 1.112 | 1.008 | 0.943 | 1.076 |
| Time spent in vehicular trips | **0.981** | **0.968** | **0.993** | **0.985** | **0.976** | **0.994** |
| Time spent in pedestrian trips | 0.991 | 0.967 | 1.017 | 0.987 | 0.972 | 1.004 |
| Sub-optimal compliance (vs. optimal compliance) | 0.933 | 0.662 | 1.316 | 0.865 | 0.681 | 1.099 |
| Worst compliance (vs. optimal compliance) | **0.446** | **0.311** | **0.640** | **0.624** | **0.489** | **0.796** |
| Missing (NA) compliance (vs. optimal compliance) | 1.048 | 0.179 | 6.150 | 0.864 | 0.293 | 2.547 |
| Outdoor temperature | **0.954** | **0.919** | **0.990** | **0.974** | **0.950** | **0.998** |

Significant associations are highlighted in bold.
